# Chromosomal abnormalities and atrial fibrillation and ischemic stroke incidence: a nationwide population-based study

**DOI:** 10.1038/s41598-020-72678-0

**Published:** 2020-09-28

**Authors:** Jun Hwan Cho, Eue-Keun Choi, In-Ki Moon, Jin- Hyung Jung, Kyung-Do Han, You-Jung Choi, Jiesuck Park, Euijae Lee, So- Ryoung Lee, Myung-Jin Cha, Woo-Hyun Lim, Seil Oh

**Affiliations:** 1 Department of Internal Medicine, Seoul National University Hospital, 101 Daehak-ro, Jongno-gu, Seoul, 03080 Republic of Korea; 2 Heart Research Institute, Cardiovascular-Arrhythmia Center, College of Medicine, Chung-Ang University Hospital, Seoul, Republic of Korea; 3 Department of Biostatistics, College of Medicine, The Catholic University of Korea, Seoul, Republic of Korea; 4 Department of Internal Medicine, Seoul National University Boramae Medical Center, Seoul, Republic of Korea

**Keywords:** Atrial fibrillation, Risk factors

## Abstract

There is a paucity of information as to whether chromosomal abnormalities, including Down Syndrome, Turner Syndrome, and Klinefelter Syndrome, have an association with atrial fibrillation (AF) and ischemic stroke development. Data from 3660 patients with Down Syndrome, 2408 with Turner Syndrome, and 851 with Klinefelter Syndrome without a history of AF and ischemic stroke were collected from the Korean National Health Insurance Service (2007–2014). These patients were followed-up for new-onset AF and ischemic stroke. Age- and sex-matched control subjects (at a ratio of 1:10) were selected and compared with the patients with chromosomal abnormalities. Down Syndrome patients showed a higher incidence of AF and ischemic stroke than controls. Turner Syndrome and Klinefelter Syndrome patients showed a higher incidence of AF than did the control group, but not of stroke. Multivariate Cox regression analysis revealed that three chromosomal abnormalities were independent risk factors for AF, and Down Syndrome was independently associated with the risk of stroke. In conclusion, Down Syndrome, Turner Syndrome, and Klinefelter Syndrome showed an increased risk of AF. Down Syndrome patients only showed an increased risk of stroke. Therefore, AF surveillance and active stroke prevention would be beneficial in patients with these chromosomal abnormalities.

## Introduction

Down Syndrome, Klinefelter Syndrome, and Turner Syndrome constitute the most common chromosomal abnormalities. Down Syndrome is the most common chromosomal abnormality worldwide, with an incidence of approximately 1:700 live births^1^. This syndrome is typically caused by trisomy of chromosome 21. Klinefelter Syndrome is the most common sex-chromosome disorder in men with a prevalence of approximately 1:600 men^2^, and is defined as men having a karyotype containing an extra X-chromosome (47, XXY) due to mosaicism^3^. Turner Syndrome is the most common sex-chromosome abnormality in women with a prevalence of approximately 1:2000 women^4^, and is characterized by complete or partial X chromosome monosomy.

Since the first report of these chromosomal abnormalities, life expectancy has markedly increased due to the development of medical care. In Down Syndrome, the mean life expectancy at age 12 years has increased to approximately 60 years^5,6^. Furthermore, recent studies have shown that the median survival age in patients with Klinefelter Syndrome has increased to 71.4 years^7^. As the average life expectancy of people with chromosomal abnormalities increases, more attention should be paid to the age-related chronic disorders that may occur in these patients. Thus, there is a greater need for medical care of people with chromosomal abnormalities to prevent and treat age-related chronic disorders. In the general population, atrial fibrillation (AF) and ischemic stroke are prevalent with increasing age^8,9^. However, there is paucity information as to whether these chromosomal abnormalities are associated with AF and ischemic stroke development.

Therefore, this study aimed to evaluate the association between the three most common and representative chromosomal abnormalities and the incidence of AF and ischemic stroke in a population-based cohort study using the National Health Insurance Service (NHIS) database in Korea.

## Methods

### Data sources and study patients

This study used the NHIS database. The NHIS is a mandatory health insurance program managed by the Korean government that covers the majority (97%) of the Korean population. Records from the NHIS database include sociodemographic information, medical treatment, and disease diagnosed according to the International Classification of Disease-10 (ICD-10)^10^.

The NHIS is administrated by the Korean government and operates the Rare Intractable Disease (RID) programs that offer financial support to patients who have a certain rare or intractable disease. The RID program requires the physician to complete the application for registration in patients who have been diagnosed with Down Syndrome, Klinefelter Syndrome, and Turner Syndrome confirmed by genetic testing. The application contains detailed diagnostic methods used and the name of the physician who confirmed the diagnosis and license number. After reviewing the application, a RID code is provided by the NHIS to certify their diagnosis of Down Syndrome (V159), Klinefelter Syndrome (V218), or Turner Syndrome (V021).

To protect the individual information, resident registration numbers were encrypted. The database is open to all researchers whose study protocols have been approved by the official review committee. This study was exempt from review by the Seoul National University Hospital Institutional Review Board (E-1711-081-900).

Based on the claimed data, 49,570,064 patients were recorded in 2007. Patients with Down Syndrome, Klinefelter Syndrome, and Turner Syndrome were selected from the NHIS sample cohort during the screening period from January 1, 2007, to December 31, 2014. The definition of each chromosomal abnormality on ICD-10 codes and RID code were as follows: Down Syndrome (Q90.0–Q90.2, Q90.9, V159), Klinefelter Syndrome (Q98.0–Q98.9, V218), and Turner Syndrome (Q96.0–Q96.4, Q96.8, Q96.9, V021).

Patients diagnosed with AF and/or ischemic stroke during the screening period were excluded and a total of 3660 patients with Down Syndrome, 851 with Klinefleter Syndrome, and 2408 with Turner Syndrome were identified. For comparison, 1:10 age and sex-matched controls without these chromosomal abnormalities were selected for each chromosomal abnormality as a control group. All patients or subjects in the control group were followed-up until December 31, 2014. The average follow-up period was 5.9 ± 2.4 years in Down Syndrome, 5.7 ± 2.4 years in Klinefelter Syndrome, and 6.8 ± 2.5 years in Turner Syndrome.

### Defining outcomes and comorbidities

The primary end-point was the development of newly diagnosed non-valvular AF and ischemic stroke. Non-valvular AF was defined using ICD-10 codes I48.0–I48.4 and I48.9. Either a diagnosis during hospitalization or more than two diagnoses at outpatient clinics were required to diagnose AF^8,11^. Individuals diagnosed with mitral stenosis (I05.0–I05.2 and I05.9) or those with mechanical heart valves (Z95.2–Z95.4) were excluded, as in our previous study^8^. Ischemic stroke was defined using ICD-10 codes I63–I64 given during hospitalization combined with claims for neurological imaging using computed tomography or magnetic resonance imaging^11^.

Comorbidities including hypertension, diabetes mellitus (DM), dyslipidemia, chronic obstructive pulmonary disease (COPD), ischemic heart disease (IHD), chronic heart failure (CHF), end-stage renal disease (ESRD), and peripheral arterial disease (PAD) were also defined using the ICD-10 codes. The definitions of outcomes and comorbidities are presented in Supplementary Table 1. Low income was defined as the lowest 20% of the total population based on the individual’s monthly income.

### Statistical analysis

Categorical variables are presented as numbers and relative frequencies (percentages) and were compared using the Chi-squared test. Continuous variables are expressed as mean ± standard deviation and analyzed using the Student’s *t* test. Comparison of cumulative event rates between the three chromosomal abnormalities were based on Kaplan–Meier censoring estimates and compared using the log-rank test. The incidence rate of AF and stroke were described as the number of events per 1000 person-years. Hazard ratios (HR) and the corresponding 95% confidence intervals (CI) were calculated using Cox proportional hazard models. To investigate the association between the chromosomal abnormalities and AF, the multivariate Cox regression model was adjusted for age, sex, income, DM, hypertension, dyslipidemia, COPD, IHD, CHF, ESRD, and PAD. Subgroup analyses divided by multiple cardiovascular (CV) risk factors were subsequently performed. All *P* values were two-sided, and a value of less than 0.05 was considered statistically significant. Statistical analyses were performed using SAS version 9.3 (SAS Institute, Cary, NC, USA).

## Results

### Baseline characteristics of the cohort

The baseline characteristics of the study population are summarized in Table 1. In patients with Down Syndrome, the mean age of the study participants was 10.0 ± 11.9 years, and 55.8% were men. Most patients with Down Syndrome were aged ≤ 19 years (82.7%; 3028 with Down Syndrome and 30,280 in the control group); 16.5% were aged 20–49 years (603 with Down Syndrome and 6030 in the control group), and 0.8% were aged ≥ 50 years (29 with Down Syndrome and 290 in the control group). Patients with Down Syndrome had a higher rate of comorbidities such as hypertension, DM, dyslipidemia, COPD, IHD, CHF, ESRD, and PAD compared to the control group.

**Table 1:** Baseline characteristics of the study population.

Characteristics	Down Syndrome			Klinefelter Syndrome			Turner Syndrome		
	No (n = 36,600)	Yes (n = 3660)	*P* value	No (n = 8510)	Yes (n = 851)	*P* value	No (n = 24,080)	Yes (n = 2408)	*P* value
Male (%)	20,420 (55.79)	2042 (55.79)	1	8510 (100%)	851 (100%)	1	–	–	–
Female (%)	16,180 (44.21)	1618 (44.21)	1	–	–	–	24,080 (100%)	2408 (100%)	1
Age (years)	9.98 ± 11.91	9.98 ± 11.91	1	29.16 ± 13.54	29.16 ± 13.55	1	19.18 ± 11.02	19.18 ± 11.02	1
≤ 19	30,280 (82.73%)	3028 (82.73%)	1	1930 (22.68%)	193 (22.68%)	1	14,040 (58.31%)	1404 (58.31%)	1
20–49	6030 (16.48%)	603 (16.48%)		6230 (73.21%)	623 (73.21%)		9800 (40.7%)	980 (40.7%)	
≥ 50	290 (0.79%)	29 (0.79%)		350 (4.11%)	35 (4.11%)		240 (1.00%)	24 (1.00%)	
Low income*	6515 (17.8%)	1183 (32.32%)	< 0.0001	1680 (19.74%)	159 (18.68%)	0.4591	5296 (21.99%)	606 (25.17%)	0.0004
Hypertension	178 (0.49%)	130 (3.55%)	< 0.0001	310 (3.64%)	34 (4.00%)	0.6023	159 (0.66%)	81 (3.36%)	< 0.0001
Diabetes mellitus	80 (0.22%)	74 (2.02%)	< 0.0001	119 (1.4%)	61 (7.17%)	< 0.0001	83 (0.34%)	115 (4.78%)	< 0.0001
Dyslipidemia	134 (0.37%)	47 (1.28%)	< 0.0001	209 (2.46%)	72 (8.46%)	< 0.0001	84 (0.35%)	76 (3.16%)	< 0.0001
COPD	890 (2.43%)	213 (5.82%)	< 0.0001	212 (2.49%)	49 (5.76%)	< 0.0001	529 (2.20%)	91 (3.78%)	< 0.0001
IHD	86 (0.23%)	75 (2.05%)	< 0.0001	84 (0.99%)	24 (2.82%)	< 0.0001	78 (0.32%)	36 (1.5%)	< 0.0001
CHF	20 (0.05%)	175 (4.78%)	< 0.0001	15 (0.18%)	4 (0.47%)	0.0694	14 (0.06%)	18 (0.75%)	< 0.0001
ESRD	1 (0%)	10 (0.27%)	< 0.0001	9 (0.11%)	2 (0.24%)	0.294	6 (0.02%)	1 (0.04%)	0.6325
PAD	39 (0.11%)	13 (0.36%)	< 0.0001	66 (0.78%)	9 (1.06%)	0.3789	64 (0.27%)	5 (0.21%)	0.5936
Duration (stroke)	5.9 ± 2.4	5.9 ± 2.4	0.7012	5.7 ± 2.4	5.7 ± 2.4	0.9949	6.8 ± 2.5	6.8 ± 2.5	0.9813
Duration (AF)	5.9 ± 2.4	5.9 ± 2.4	0.5152	5.7 ± 2.4	5.7 ± 2.4	0.8249	6.8 ± 2.5	6.8 ± 2.5	0.8296
Number of PY (stroke)	216,494.5	21,591.3		48,877.7	4888.2		163,227.3	16,325.7	
Number of PY (AF)	216,416.2	21,542.8		48,847.3	4868.8		163,188.9	16,291.6	

*Denotes subjects with an annual income lower than 20% among the total population.

*COPD* chronic obstructive pulmonary disease, *IHD* ischemic heart disease, *CHF* chronic heart failure, *ESRD* end-stage renal disease, *PAD* peripheral arterial disease, *AF* atrial fibrillation, *PY* patients-years.

In patients with Klinefelter Syndrome and Turner Syndrome, the mean age was 29.2 ± 13.5 and 19.2 ± 11.0 years, respectively. Both groups had a higher incidence of comorbidities, such as DM, dyslipidemia, COPD, and IHD than the control groups. In Turner Syndrome, the prevalence of hypertension and CHF were higher than in the control group, unlike in Klinefelter Syndrome. In Down Syndrome and Turner Syndrome, the rate of patients with low income was higher than those in each of the control groups (*P* < 0.001 and *P* < 0.001, respectively), whereas Klinefelter Syndrome was not (*P* = 0.46).

### Incidence rates and relative risk of AF according to chromosomal abnormalities

During the mean follow-up of 5.9 years, new-onset AF was diagnosed in 55 patients (24 patients (0.7%) in the Down Syndrome group and 31 (0.1%) in the control group). In the Klinefelter Syndrome group, 6 patients were diagnosed with new-onset AF (0.7%), and 22 patients (0.3%) were diagnosed in the control group during a mean follow-up of 5.7 years. Seven patients (0.3%) were diagnosed as new-onset AF in the Turner Syndrome group during the 6.8 year-follow-up, whereas 22 patients (0.1%) were newly diagnosed with AF during the same follow-up period in the control group.

**Figure 1:**
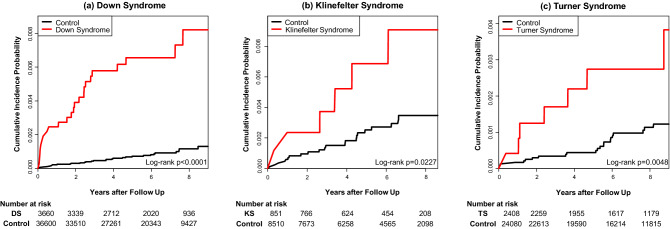
Comparison of cumulative incidence of atrial fibrillation events in chromosomal abnormalities. (**a**) Down Syndrome, (**b**) Klinefelter Syndrome (**c**) Turner Syndrome. *DS* Down Syndrome, *KS* Klinefelter Syndrome, *TS* Turner Syndrome.

The cumulative incidence of AF for each chromosomal abnormality is shown in Fig. 1. AF incidence was significantly higher for all three chromosomal abnormalities compared to each control group (*p* < 0.001 in Down Syndrome, *p* = 0.023 in Klinefelter Syndrome, *p* = 0.005 in Turner Syndrome, by log-rank test). Table 2 shows the incidence rate of AF and the crude and adjusted HRs in the three chromosomal abnormalities. All three chromosomal abnormalities showed a higher incidence of AF compared to each control group (1.114 vs. 0.143 in Down Syndrome, 1.232 vs. 0.450 in Klinefelter Syndrome, 0.430 vs. 0.135 in Turner Syndrome, in 1000 person-years (PY) respectively). All three chromosomal abnormalities showed a higher risk of AF compared to each control group with or without multivariate adjustment (adjusted HR: 6.84, 95% CI: 3.77–12.20 in Down Syndrome; adjusted HR: 2.84, 95% CI: 1.01–6.88 in Klinefelter Syndrome, and adjusted HR: 2.75, 95% CI: 1.03–6.43 in Turner Syndrome).

**Table 2:** Risk of the atrial fibrillation events in chromosomal abnormalities.

Groups	N	No. of events	Incidence rate (IR)^a^	Hazard Ratio (95% CI)	
				Crude HR^b^	Adjusted HR^c^
**Down Syndrome**					
No	36,600	31	0.143	1 (ref.)	1 (ref.)
Yes	3660	24	1.114	7.77 (4.52–13.20)	6.84 (3.77–12.20)
**Klinefelter Syndrome**					
No	8510	22	0.450	1 (ref.)	1 (ref.)
Yes	851	6	1.232	2.74 (1.00–6.34)	2.84 (1.01–6.88)
**Turner Syndrome**					
No	24,080	22	0.135	1 (ref.)	1 (ref.)
Yes	2408	7	0.430	3.19 (1.26–7.10)	2.75 (1.03–6.43)

*HR* hazard ratio, *CI* confidence interval, *COPD* chronic obstructive pulmonary disease, *IHD* ischemic heart disease, *CHF* Chronic heart failure, *ESRD* End-stage renal disease, *PAD* peripheral artery disease.

^a^Incidence rates were calculated per 1000 patient-years.

^b^Unadjusted crude hazard ration (HR) and 95% CI.

^c^Multivariate Cox regression model adjusted for age, sex, income, diabetes mellitus, hypertension, dyslipidemia, COPD, IHD, CHF, ESRD, and PAD.

### Incidence rates and relative risk of ischemic stroke according to chromosomal abnormalities

**Figure 2:**
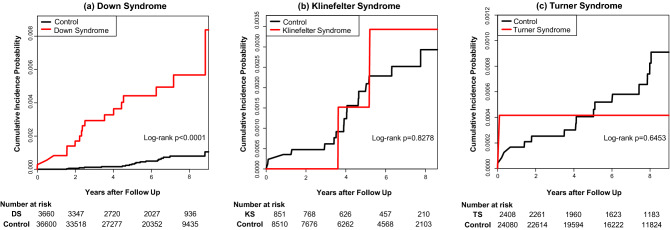
Comparison of cumulative incidence of ischemic stroke events in chromosomal abnormalities. (**a**) Down Syndrome, (**b**) Klinefelter Syndrome (**c**) Turner Syndrome. *DS* Down Syndrome, *KS* Klinefelter Syndrome, *TS* Turner Syndrome.

During the mean follow-up of 5.9 years, 17 patients (0.5%) in the Down Syndrome group and 19 (0.1%) in the control group were newly diagnosed with ischemic stroke. During the mean follow-up of 5.7 years, 2 patients (0.2%) experienced stroke in the Klinefelter Syndrome group and 17 (0.2%) in the control group. In the Turner Syndrome group, one patient (0.04%) was newly diagnosed with stroke, whereas in the control group, 16 patients (0.07%) were newly diagnosed with stroke during a same mean follow-up of 6.8 years. There was a higher incidence of stroke and a higher cumulative incidence of stroke in the Down Syndrome group compared to the control group (Fig. 2). However, the cumulative incidence of stroke in the Klinefelter Syndrome and Turner Syndrome groups was not significantly different as compared to each control group (*p* = 0.82 in Klinefelter Syndrome, *p* = 0.65 in Down Syndrome). The risk of stroke events in each of the chromosomal abnormalities is summarized in Table 3. In Down Syndrome, the risk of stroke was higher in the crude and adjusted HR compared to the control group (crude HR: 8.98, 95% CI: 4.62–17.30, adjusted HR: 7.36, 95% CI: 3.48–15.35). However, both Klinefelter Syndrome and Turner Syndrome groups did not show statistical significance.

**Table 3:** Risk of the ischemic stroke events in chromosomal abnormalities.

Groups	N	No. of events	Incidence rate (IR)^a^	Hazard ratio (95% CI)	
				Crude HR^b^	Adjusted HR^c^
**Down Syndrome**					
No	36,600	19	0.088	1 (ref.)	1 (ref.)
Yes	3660	17	0.787	8.98 (4.62–17.30)	7.36 (3.48–15.35)
**Klinefelter Syndrome**					
No	8510	17	0.348	1 (ref.)	1 (ref.)
Yes	851	2	0.409	1.18 (0.19–4.10)	1.57 (0.25–5.60)
**Turner Syndrome**					
No	24,080	16	0.098	1 (ref.)	1 (ref.)
Yes	2408	1	0.061	0.63 (0.04–3.06)	0.38 (0.02–2.07)

*HR* hazard ratio, *CI* confidence interval, *COPD* chronic obstructive pulmonary disease, *IHD* ischemic heart disease, *CHF* chronic heart failure, *ESRD* end-stage renal disease, *PAD* peripheral artery disease.

^a^Incidence rates were calculated per 1000 patient-years.

^b^Unadjusted crude hazard ration (HR) and 95% CI.

^c^Multivariate Cox regression model adjusted for age, sex, income, diabetes mellitus, hypertension, dyslipidemia, COPD, IHD, CHF, ESRD, and PAD.

### Subgroup analyses for the risk of AF in chromosomal abnormalities

**Figure 3:**
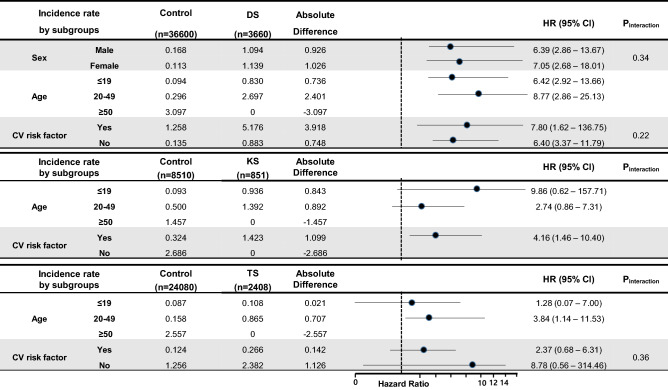
Subgroup analyses for risk of atrial fibrillation in chromosomal abnormalities. *DS* Down Syndrome, *KS* Klinefelter Syndrome, *TS* Turner Syndrome, *CV risk factor* patients who have either hypertension, diabetes or dyslipidemia.

Subgroup analyses for the risk of AF in each chromosomal abnormality are shown in Fig. 3. The Down Syndrome group consistently demonstrated a higher incidence rate and risk of AF development compared to the control group in all subgroups. Regarding the effect of age on AF in the Down Syndrome group, younger age showed a stronger association with AF, as did the presence of a well-known cardiovascular risk factor. No AF development occurred in patients with Down Syndrome aged over 50 years. In Klinefelter Syndrome, no significant difference was observed in the subgroup analyses by age. However, the risk of AF in Klinefelter Syndrome patients with cardiovascular risk factors was higher than in the control group (HR: 4.16, 95% CI: 1.46–10.40). In Turner Syndrome, no significant difference was observed in the subgroup analyses, unlike in Down Syndrome.

### Subgroup analyses on the risk of ischemic stroke in chromosomal abnormalities

**Figure 4:**
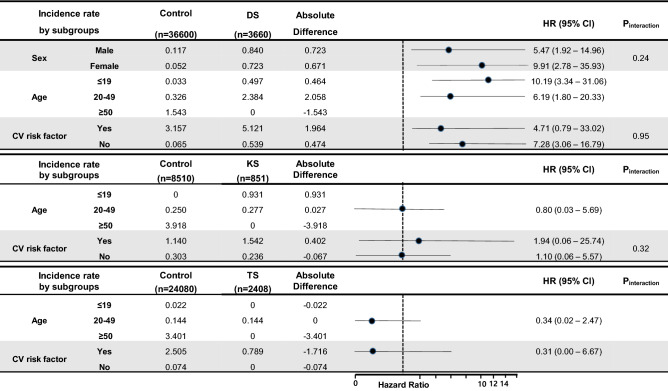
Subgroup analyses for risk of ischemic stroke in chromosomal abnormalities. *DS* Down Syndrome, *KS* Klinefelter Syndrome, *TS* Turner Syndrome; *CV risk factor* patients who have either hypertension, diabetes or dyslipidemia.

Subgroup analyses on the risk of ischemic stroke in each chromosomal abnormality are shown in Fig. 4. Patients with Down Syndrome showed a consistently higher risk of ischemic stroke according to age, sex, and cardiovascular risk factor subgroups. Female patients with Down Syndrome showed a 9.91-fold higher risk of ischemic stroke compared to those without Down Syndrome, which is higher than that of male patients with Down Syndrome.

In the age subgroups, patients with Down Syndrome aged 20–49 years showed a higher incidence of stroke compared to those aged under 19 years. The effect of Down Syndrome on stroke development was relatively higher in the subgroups of patients with childhood Down Syndrome compared to those of middle-aged patients. No patients with Down Syndrome were newly diagnosed with stroke aged over 50 years. The effect of Down Syndrome on ischemic stroke was stronger in older aged patients and those with a well-known cardiovascular risk factor. However, Klinefelter Syndrome and Turner Syndrome did not show any significant differences compared to controls in the subgroup analyses.

## Discussion

This study investigated the risk of AF and ischemic stroke in patients with chromosomal abnormalities using a nationwide population database. To the best of our knowledge, this is the first study reporting the risk of AF and ischemic stroke in well-known chromosomal abnormalities. This study showed three important findings: (1) AF incidence was higher in all three chromosomal abnormalities compared to the matched population and all three chromosomal abnormalities showed a higher risk of AF, (2) Down Syndrome showed a higher risk of ischemic stroke, whereas Klinefelter Syndrome and Turner Syndrome did not, and (3) in people with chromosomal disease, cardiovascular disease develops at a relatively young age; the long duration of cardiovascular disease leads to an increase in the incidence of AF and stroke as a common pathway.

### Risk of AF and ischemic stroke in Down Syndrome

The risk of cardiovascular events in patients with Down Syndrome was reported in a previous study^12^. In this study, the prevalence of diabetes, sleep apnea, and pulmonary hypertension was higher in patients with Down Syndrome. In addition, the prevalence of diabetes and hypertension increased in older patients with Down Syndrome. The rate of a cardiovascular risk factor (defined as presence of any of congenital heart disease, cardiac arrhythmia, pulmonary hypertension, hypertension, diabetes, sleep apnea, smoking, or moyamoya disease) was 35.9% in patients with Down Syndrome and 19.2% in the control group. The risk of ischemic stroke is higher in patients with Down Syndrome, which has a high cardioembolic risk factor (defined as either congenital heart disease, cardiac arrhythmia, or pulmonary hypertension). However, the prevalence of any coronary event and myocardial infarction were lower compared to the control group^12^. These results show the same trend as reported in other studies^13–15^.

The prevalence of cardiac arrhythmia (2%) was also higher in patients with Down Syndrome compared to those without Down Syndrome. The incidence rate of any cardiac arrhythmia was higher in patients with Down Syndrome aged under 19 years and 20–50 years, but the absolute risk difference was higher in adolescents (0.8% in Down Syndrome vs. 0.04% in controls, 1.8% in Down Syndrome vs. 0.9% in controls, respectively). However, this study analyzed all types of arrhythmia including ventricular arrhythmia, sick sinus syndrome, and supraventricular arrhythmia, as well as AF. No study has focused on the association between Down Syndrome and AF. We found that the incidence of AF was higher in patients with Down Syndrome, showing an increased risk of developing AF compared to those without Down Syndrome after adjusting for various cardiovascular comorbidities.

Several plausible explanations on the mechanism of AF in patients with Down Syndrome are available. In line with a previous study^12^, the incidence of traditional cardiovascular risk factors was high for AF development in patients with Down Syndrome. However, the risk of AF in patients with Down Syndrome was independently higher after adjusting for cardiovascular comorbidities. Second, patients with Down Syndrome are known to have a higher risk of congenital heart disease, which was frequently combined with atrial tachycardia or AF^16,17^. Moreover, postoperative scar and intra-atrial conduction delay might increase the risk of AF^18,19^. Third, altered autonomic cardiac regulation might also contribute to the risk of AF. Patients with Down Syndrome and a structurally normal heart had a lower resting heart rate and decreased blood pressure responses to exercise, as well as reduced heart rate recoveries after exercise. These abnormalities are attributed to inadequate sympathetic activation, and prominent vagal modulation found even in patients with Down Syndrome and structurally normal hearts^20,21^. This autonomic dysregulation plays an important role in the pathogenesis of AF^22,23^. Lastly, increased P wave dispersion was observed in patients with Down Syndrome^24^, reflecting the tendency of AF and potential substrate for AF^25^.

Regarding the risk of cerebrovascular complications, a previous study reported that Down Syndrome was associated with a high risk of stroke^12^. In line with a previous study, we found that the risk of stroke was increased in patients with Down Syndrome compared to those without. Interestingly, female and middle-aged patients with Down Syndrome are at a higher risk of stroke. The association between Down Syndrome and stroke could be explained as follows. First, the increased risk of AF in patients with Down Syndrome could contribute to an increased risk of stroke in this population. Second, patients with Down Syndrome are known to have highly prevalent hypothyroidism with subclinical hypothyroidism present in up to 25–60% of patients^26,27^, which could increase the risk of stroke^28^. Lastly, patients with Down Syndrome have anatomic cerebral vessel peculiarities, such as those described in moyamoya disease. Moyamoya disease is prevalent in patients with Down Syndrome^12,29^, and patients with Down Syndrome account for 8.7% of all patients with moyamoya disease in a recent large cohort study^29^.

### Risk of AF and ischemic stroke in Klinefelter Syndrome

Klinefelter Syndrome is the most common male sex chromosomal disorder^2^, with features including small testes, azoospermia, and increased LH and FSH^30,31^. Two large national epidemiological studies in Denmark and Britain have reported the increased risk of cardiovascular mortality in patients with Klinefelter Syndrome. In the Danish cohort study, the hazard ratio (HR) of mortality due to cardiovascular disease was 1.4 (95% CI: 1.03–1.93)^32^. In the British cohort study, the standard mortality rate (SMR) of cardiovascular disease was 1.3 (95% CI: 1.1–1.5). In addition, the SMR of pulmonary embolism and other heart disease were 5.7 (95% CI: 2.5–11.3) and 2.2 (95% CI: 1.3–3.6), respectively. However, the SMR of ischemic heart disease was 0.7 (95% CI: 0.5–0.9)^33^. However, neither study reported an association between AF and Klinefelter Syndrome. Although cardiovascular risk factors were more prevalent in patients with Klinefelter Syndrome, the risk of AF was found to be increased after adjusting for several cardiovascular risk factors. Interestingly, the risk of AF was increased especially in adolescents and patients with a cardiovascular risk factor.

Generally, Klinefelter Syndrome diagnosis is purely dependent on a clinical suspicion based on commonly subtle clinical symptoms. Only 25% of cases are diagnosed at an early stage and in most cases, diagnosis is not possible until after late puberty, which results in delaying testosterone hormone treatment^2^. Due to the lack of androgen, patients with Klinefelter Syndrome developed abdominal obesity followed by insulin resistance, finally causing metabolic syndrome and DM^31^. Moreover, male patients with isolated AF had lower testosterone levels compared with controls^34^. Therefore, the lack of androgen could be one of the explanations for the causal relationship between Klinefelter Syndrome and AF. Second, diastolic dysfunction on echocardiography in patients with Klinefelter Syndrome might increase the risk of AF. Patients with Klinefelter Syndrome are known to have decreased diastolic function^35,36^. Diastolic dysfunction was correlated with the free testosterone level in patients with Klinefelter Syndrome^36^ and has been well established to be strongly associated with AF^37^. Lastly, the increasing incidence of AF in aging men could partially be attributable to decreasing androgen levels^38^.

In the British cohort study, cerebrovascular disease mortality was significantly increased in patients with Klinefelter Syndrome (SMR: 2.2; 95% CI: 1.6–3.0)^39^. However, whether the cause of cerebrovascular disease is ischemic or hemorrhagic remains unclear. Price et al. reported that the rupture of a berry aneurysm was the third most common cause of death in middle-aged patients with Klinefelter Syndrome^40^. Our study showed that Klinefelter Syndrome was not an independent risk factor for ischemic stroke. Although Klinefelter Syndrome is associated with a high risk of venous thromboembolism and thrombotic risk^41^, it is thought to be a secondary cause of DM and metabolic disease rather than a direct association with Klinefelter Syndrome.

### Risk of AF and ischemic stroke in Turner Syndrome

Cardiovascular morbidity and mortality in patients with Turner Syndrome were higher than that in the control group in both the Danish and British cohorts. In the Danish cohort study, women with Turner Syndrome had an increased risk of cardiovascular disease compared to the general female population^42^. Moreover, the risk of cardiovascular in patients with Turner Syndrome was increased up to four times than that in the British cohort study^43^. Women with Turner Syndrome have multiple combined cardiovascular risk factors, including hypertension, obesity, DM, and dyslipidemia. Hypertension has been reported in half of adults and a quarter of adolescents with Turner Syndrome^44^. Obesity and dyslipidemia are frequently combined, and the risk of DM development is fourfold higher in patients with Turner Syndrome^42,45^. Moreover, IHD is frequently observed in patients with Turner Syndrome^4^. In line with previous studies, the prevalence of hypertension, DM, dyslipidemia, IHD, and HF was found to be higher in patients with Turner Syndrome.

However, no previous study has investigated the association between Turner Syndrome and AF. The incidence of AF was higher in patients with Turner Syndrome and more than threefold higher than in all patients. In addition, Turner Syndrome showed a higher risk of AF.

In patients with Turner Syndrome, heart rate variability is high and P-wave dispersion and inhomogeneous atrial depolarization are well known^46^. These phenomenon increases the atrial arrhythmogenic potential and can cause AF^47^.

In both the British and Danish cohorts, the incidence of stroke increased; morbidity (RR: 2.7) and mortality (SMR: 3.9) also increased^42,43^. However, the etiology of stroke is uncertain, an estimated 90% of events were hemorrhagic in one cohort^43^. Only a few case reports have demonstrated individual causes of ischemic stroke in patients with Turner Syndrome^48,49^. In our study, the incidence of ischemic stroke in Turner Syndrome patients was not higher than that in the control group, and Turner Syndrome itself was not found to be an independent factor for developing ischemic stroke. Further studies on the association between Turner Syndrome and ischemic stroke are needed.

We found that AF incidence was higher in all three chromosomal abnormalities. All three chromosomal abnormalities have a high incidence of cardiovascular risk factors such as DM, hypertension, obesity, and metabolic syndrome, which are known risk factors of AF. However, after adjusted with these comorbidities, all three chromosomal abnormalities showed a higher risk of AF. The potential mechanism of chromosomal abnormalities on the risk of AF could be explained as dysregulation of the sympathetic nervous system, leading to tachycardia and increasing heart rate variation, and P-wave dispersion^20–25,35,36,46,47^. However, each chromosomal disease has a different phenotype and clinical characteristics, especially for cardiovascular disease, so the detailed mechanism of each chromosomal abnormality on AF development would be studied in the future. Down syndrome only showed a higher risk of ischemic stroke, whereas Klinefelter and Turner syndromes did not. The low incidence of ischemic stroke and a relatively small number of population in these two chromosomal abnormalities would be a plausible reason for statistical insignificance. Also, we need to study the mechanism of a higher risk of ischemic stroke in chromosomal abnormalities in the future*.* Considering the high risk of AF at a relatively young age, active AF surveillance and stroke prevention should be important, especially in adolescents with chromosomal abnormalities.

Our study has several limitations. First, this is a nationwide population-based observational study that is susceptible to several biases, including selection bias. Second, because comorbidities were identified using diagnostic codes included in the claims data, this relies on the assumption that the physician entered the correct diagnosis for each patient. Third, we did not adjust for other confounders not included in the claims database, such as obesity, history of cardiac surgery, etc. Fourth, additional information regarding ischemic stroke, such as results of brain MRI or etiology work-up, were not available in the Korean NHIS database. Therefore, the cause of ischemic stroke in this study could not be validated, which is an inevitable limitation of the study using claims data. Fifth, the majority of our study population was relatively young, so the data on patients aged over 50 years was limited. More cases in patients aged over 50 years old with each chromosomal abnormalities are needed to support our observation.

## Conclusion

Although the incidence of AF is higher in all three chromosomal abnormalities, Down Syndrome and Klinefelter Syndrome are associated with a higher risk of AF development, whereas Turner Syndrome is not. Regarding stroke, only Down Syndrome showed an increased risk of stroke, whereas Klinefelter Syndrome and Turner Syndrome did not. Therefore, a detailed transition strategy of AF surveillance and stroke prevention are important in adolescents with chromosomal abnormalities.

## Supplementary information

Supplementary Information 1.
