# Supplementary material for: Chromosomal abnormalities and atrial fibrillation and ischemic stroke incidence: a nationwide population-based study
Source: Sci Rep. 2020 Sep 28;10:15872. doi: 10.1038/s41598-020-72678-0 (PMC7522243; doi:10.1038/s41598-020-72678-0)
Supplement: Supplementary file 1 — Supplementary Information 1. [file 41598_2020_72678_MOESM1_ESM.docx]

**Chromosomal abnormalities and atrial fibrillation and ischemic stroke incidence: A nationwide population-based study**

Jun Hwan Cho^1,2^, Eue-Keun Choi^1^, In-Ki Moon^1^, Jin-Hyung Jung^3^, Kyung-Do Han^3^, You-Jung Choi^1^, Jiesuck Park^1^, Euijae Lee^1^, So-Ryoung Lee^1^, Myung-Jin Cha^1^, Woo-Hyun Lim^4^, Seil Oh^1^

^1^Department of Internal Medicine, Seoul National University Hospital, Seoul, Republic of Korea;

^2^Heart Research Institute, Cardiovascular-Arrhythmia Center, College of Medicine, Chung-Ang University Hospital, Seoul, Republic of Korea;

^3^Department of Biostatistics, College of Medicine, The Catholic University of Korea, Seoul, Republic of Korea;

^4^Department of Internal Medicine, Seoul National University Boramae Medical Center, Seoul, Republic of Korea

**These authors take responsibility for all aspects of the reliability and freedom from bias of the data presented and their discussed interpretation.**

**Conflict of interest statement**

All authors declare that there is no conflict of interest relevant to the submitted work.

**Correspondence**:

Eue-Keun Choi, MD, PhD

Department of Internal Medicine, Seoul National University Hospital

101 Daehak-ro, Jongno-gu, Seoul, 03080, Republic of Korea

Tel: (82)-2-2072-0688, Fax : (82)-2-762-9662

E-mail: [choiek17@snu.ac.kr](mailto:choiek17@snu.ac.kr)

**Supplementary Table 1.** List of the definitions of comorbidities and outcome

|  | ICD-10 codes | Additional definitions |
| --- | --- | --- |
| **Comorbidities** |  |  |
| Hypertension | I10-I15 | Hospitalization ≥ 1 or Outpatient visit ≥ 2 |
| Diabetes mellitus | E11-E14 | Hospitalization ≥ 1 or Outpatient visit ≥ 2  With additional claims for the oral antidiabetics or insulin |
| Dyslipidemia | E78 | Any of hospitalization or outpatient visit ≥ 1 |
| Congestive heart failure | I50 | Any of hospitalization or outpatient visit ≥ 1 |
| Peripheral artery disease | I70, I73 | Hospitalization ≥ 1 or Outpatient visit ≥ 2 |
| COPD | J43, J44 | Hospitalization ≥ 1 or Outpatient visit ≥ 2 |
| End stage renal disease | N18-19, Z49, Z905, Z94, Z992 | Hospitalization ≥ 1 or Outpatient visit ≥ 2 |
| Ischemic heart disease | I20-25 | Hospitalization ≥ 1 or Outpatient visit ≥ 2 |
| **Outcomes** |  |  |
| Nonvalvular AF | I48.0-I48.4, I48.9 | Hospitalization ≥ 1 or Outpatient visit ≥ 2  With excluding mitral stenosis (I05.0, I05.2, I05.9) and mechanical heart valves (Z95.2-Z95.4) |
| Stroke | I63, I64 | Hospitalization ≥ 1 or Outpatient visit ≥ 2  With recorded as the diagnosis for admission with brain computed tomography or magnetic resonance imaging. |
